# IoT‐Based Elderly Health Monitoring System Using Firebase Cloud Computing

**DOI:** 10.1002/hsr2.70498

**Published:** 2025-03-02

**Authors:** Adhan Efendi, Muhammad Imam Ammarullah, Indra Griha Tofik Isa, Meli Puspita Sari, Jasmine Nurul Izza, Yohanes Sinung Nugroho, Hamid Nasrullah, Denny Alfian

**Affiliations:** ^1^ Graduate Institute of Precision Manufacturing National Chin‐Yi University of Technology Taichung Taiwan; ^2^ Department of Mechanical Engineering, Faculty of Engineering Universitas Diponegoro Semarang Central Java Indonesia; ^3^ Undip Biomechanics Engineering & Research Centre (UBM‐ERC) Universitas Diponegoro Semaran Central Java indonesia; ^4^ Department of Informatics Management Politeknik Negeri Sriwijaya Palembang South Sumatra Indonesia; ^5^ Faculty of Agro‐Industrial Technology Universitas Padjajaran Sumedang West Java Indonesia; ^6^ Department of Biology, Faculty of Mathematics and Natural Sciences Universitas Negeri Malang Malang East Java Indonesia; ^7^ Department of Aeronautical Engineering Politeknik Negeri Bandung Bandung West Java Indonesia; ^8^ Vocational Technology Education Universitas Negreri Yogyakarta Sleman Special Region of Yogyakarta Indonesia

**Keywords:** elderly health, firebase, internet of things, monitoring system, supervised learning

## Abstract

**Background and Aims:**

The increasing elderly population presents significant challenges for healthcare systems, necessitating innovative solutions for continuous health monitoring. This study develops and validates an IoT‐based elderly monitoring system designed to enhance the quality of life for elderly people. The system features a robust Android‐based user interface integrated with the Firebase cloud platform, ensuring real‐time data collection and analysis. In addition, a supervised machine learning technology is implemented to conduct prediction task of the observed user whether in “stable” or “not stable” condition based on real‐time parameter.

**Methods:**

The system architecture adopts the IoT layer including physical layer, network layer, and application layer. Device validation is conducted by involving six participants to measure the real‐time data of heart‐rate, oxygen saturation, and body temperature, then analysed by mean average percentage error (MAPE) to define error rate. A comparative experiment is conducted to define the optimal supervised machine learning model to be deployed into the system by analysing evaluation metrics. Meanwhile, the user satisfaction aspect evaluated by the terms of usability, comfort, security, and effectiveness.

**Results:**

IoT‐based elderly health monitoring system has been constructed with a MAPE of 0.90% across the parameters: heart‐rate (1.68%), oxygen saturation (0.57%), and body temperature (0.44%). In machine learning experiment indicates XGBoost model has the optimal performance based on the evaluation metrics of accuracy and F1 score which generates 0.973 and 0.970, respectively. In user satisfaction aspect based on usability, comfort, security, and effectiveness achieving a high rating of 86.55%.

**Conclusion:**

This system offers practical applications for both elderly users and caregivers, enabling real‐time monitoring of health conditions. Future enhancements may include integration with artificial intelligence technologies such as machine learning and deep learning to predict health conditions from data patterns, further improving the system's capabilities and effectiveness in elderly care.

## 1 Introduction

In recent decades, advancements in information and communication technology have been significant. One promising technological breakthrough is the internet of things (IoT), which allows various devices to connect and communicate through the internet. In the context of healthcare, IoT technology can be used to enhance the quality of life, especially for the elderly population who are vulnerable to various health conditions and require continuous monitoring [1].

The global elderly population continues to increase. According to the United Nations, by 2050, the number of people aged 60 and over is projected to reach more than two billion [2]. With the growing elderly population, the need for efficient and effective health monitoring systems becomes increasingly urgent [3]. Common health conditions faced by the elderly, such as hypertension, diabetes, heart disease, and cognitive decline, require constant monitoring and rapid response in emergency situations [4].

IoT offers a promising solution for elderly health monitoring. By utilizing various wearable devices and environmental sensors, elderly health data can be collected in real‐time and transmitted to a cloud platform for further analysis [5]. Firebase, as Google's cloud platform, provides several services that can significantly enhance the development of IoT‐based monitoring systems, including real‐time databases, authentication, and notifications [6]. These features ensure efficient data management and instant communication, making it easier to monitor and respond to health conditions as they arise.

Previous research has shown that IoT‐based health monitoring systems can improve the quality of life for the elderly by enabling early detection of potentially life‐threatening medical conditions, sending alerts to caregivers or medical personnel, and facilitating timely interventions [7]. Additionally, these systems offer peace of mind to families by allowing them to monitor their elderly relatives' health status in real‐time, thus reducing anxiety and enhancing safety [8, 9, 10].

Several studies have contributed valuable insights into the development and application of IoT in elderly health monitoring. Research by Lin et al. [11] focuses on fall detection using an inertial measurement unit (IMU) wearable device combined with smart speakers for fall verification. This system integrates IMU sensors to detect falls, while smart speakers confirm the incidents, significantly improving fall detection accuracy and reducing false alarms. The use of IoT allows real‐time data transmission to cloud platforms, ensuring immediate notification to caregivers or family members, which is crucial for timely responses in emergency situations to prevent further injuries among the elderly.

Meanwhile, Ashraf et al. [12] developed an open‐source IoT‐based health monitoring system designed to track various health parameters such as heart‐rate, body temperature, and blood oxygen levels using multiple sensors connected through an IoT network. This research highlights the low cost and ease of implementation, making the system accessible to a wide range of communities. Similarly, Waleed et al. [13] examined the challenges of managing big data in IoT‐based patient monitoring, proposing effective methods for handling and analyzing large volumes of sensor data. In another study, Rupasinghe and Maduranga [14] designed an IoT‐based elderly activity monitoring system to support ambient assisted living (AAL), with a focus on monitoring daily activities. Additionally, Nandal et al. [15] developed an IoT‐based fall detection system specifically for the elderly, providing immediate alerts to caregivers when a fall occurs.

While all these studies demonstrate the potential of IoT in enhancing elderly health and safety monitoring, each offers unique approaches tailored to specific aspects of elderly care. For instance, Lin et al.'s integration of smart speakers improves the accuracy of fall detection, whereas Ashraf et al.'s emphasis on affordability and simplicity expands the reach of IoT solutions. Waleed et al.'s focus on big data management addresses the scalability of IoT systems, which is crucial for large‐scale deployments. Collectively, these studies highlight the versatility of IoT‐based solutions in addressing the diverse needs of elderly populations, contributing to better health outcomes and improved quality of life.

Previous studies on IoT‐based elderly health monitoring systems have demonstrated various approaches and technologies used to improve the health and well‐being of the elderly. For example, Zhou et al. [16] developed an IoT‐based health monitoring system that integrates wearable sensors and environmental sensors to detect vital signs such as heart‐rate and blood pressure, as well as changes in daily activity. This system allows for real‐time collection of health data, which is then analyzed using machine learning algorithms to detect anomalies and send notifications to caregivers or medical professionals. Another study by Kumar et al. emphasized the importance of integrating wearable devices with cloud platforms to provide scalable health monitoring services. In this study, the use of cloud technology allows for large data storage and faster computational processing, which supports early detection of critical conditions in the elderly [17].

Furthermore, several studies have explored the challenges in implementing IoT‐based monitoring systems. Naranjo et al. highlighted the challenges associated with data security and privacy issues in IoT systems, which often face the risk of data breaches that can compromise patient safety [18]. Meanwhile, Li et al. focused on technical challenges, such as ensuring reliable network connectivity and reducing the power consumption of devices used by the elderly, so that the system can operate efficiently with limited power [19]. While this study demonstrates the success and benefits of an IoT‐based health monitoring system, it also identifies challenges that need to be addressed to increase the adoption and effectiveness of such systems among the elderly population.

Some of the above studies differ from the research in this paper. This article will discuss the development and implementation of an IoT‐based elderly monitoring system using Firebase cloud computing. This research will explore the system architecture, data collection and analysis methods, and evaluate the system's performance in the context of elderly health monitoring. By combining IoT technology and cloud computing, this system is expected to provide an effective and efficient solution for continuous elderly health monitoring.

## 2 Literature Review

### 2.1 IoT in Healthcare

IoT technology has been increasingly utilized in healthcare settings, offering innovative solutions for health monitoring, management, and personalized care delivery. This section provides an overview of IoT applications in healthcare, focusing on its role in enhancing elderly care and monitoring.

IoT‐based health monitoring systems leverage interconnected devices, sensors, and networks to collect and transmit real‐time health data from patients to healthcare providers. These systems enable remote monitoring of vital signs, chronic conditions, and lifestyle factors, facilitating proactive interventions and personalized care plans. Wearable devices, such as smartwatches, fitness trackers, and medical sensors, play a crucial role in capturing physiological data, including heart‐rate, blood pressure, activity levels, and sleep patterns [20].

Additionally, IoT platforms integrate data analytics and machine learning algorithms to analyze health data, identify patterns, and generate actionable insights. Cloud‐based infrastructure provides scalable storage, processing power, and connectivity for seamless data transmission and analysis. Mobile applications and web interfaces empower patients and caregivers to access health information, receive alerts, and communicate with healthcare professionals remotely [21].

Recent advancements in IoT technology have spurred innovative solutions for elderly care and aging‐in‐place initiatives. IoT‐enabled smart home systems integrate ambient sensors, smart appliances, and assistive devices to create age‐friendly living environments. These systems monitor daily activities, detect emergencies (such as falls), and provide reminders for medication adherence and routine tasks [22].

Moreover, wearable IoT devices tailored for elderly users offer features such as GPS tracking, emergency call buttons, and fall detection algorithms. These devices provide continuous monitoring and support independent living while ensuring rapid response in case of emergencies. Advanced IoT applications also incorporate predictive analytics and predictive modeling to anticipate health deterioration and trigger preventive interventions [23].

In summary, IoT technology holds immense potential to revolutionize healthcare delivery, particularly in elderly care and monitoring. By leveraging interconnected devices, data analytics, and cloud computing, IoT‐based solutions offer personalized, proactive, and efficient healthcare services for aging populations.

### 2.2 Cloud Computing in IoT

Cloud computing plays a pivotal role in augmenting the functionalities of IoT systems, offering scalability, flexibility, and reliability. By leveraging cloud infrastructure, IoT devices can offload computationally intensive tasks, such as data storage, processing, and analytics, to remote servers [24]. This capability enables IoT solutions to handle large volumes of data generated by interconnected devices, ensuring seamless operation and efficient resource utilization.

Firebase, as a cloud computing platform provided by Google, offers numerous advantages for IoT applications. First, Firebase provides real‐time database services, enabling IoT devices to synchronize data instantaneously across multiple clients and platforms [25]. This feature is particularly valuable for applications requiring timely data updates, such as sensor readings and device status notifications. Additionally, Firebase offers built‐in authentication and authorization mechanisms, ensuring secure access control for IoT devices and users [26]. This enhances the integrity and confidentiality of data transmitted and stored in the cloud, mitigating potential security risks associated with IoT deployments.

In summary, cloud computing, particularly platforms like Firebase, empowers IoT applications with scalability, real‐time data processing, and robust security features. By leveraging cloud services, IoT systems can deliver enhanced functionalities and ensure seamless operation in diverse deployment scenarios.

### 2.3 Existing Elderly Monitoring Systems

Various existing systems and technologies cater to elderly monitoring, offering diverse features, strengths, and limitations. These systems typically integrate wearable devices, ambient sensors, and communication technologies to monitor the health and activities of elderly individuals [27].

In reviewing existing systems, it's evident that there's a wide range of approaches and technologies employed. Some systems focus on specific health parameters, such as vital signs monitoring or fall detection, while others provide comprehensive solutions for overall health and well‐being monitoring [23]. Additionally, advancements in machine learning and artificial intelligence have enabled systems to analyze collected data more intelligently, providing actionable insights and predictive capabilities [28].

Despite the diversity in features and functionalities, existing elderly monitoring systems face several common challenges. These include issues related to data privacy and security, interoperability among different devices and platforms, and usability for both elderly users and caregivers. Moreover, the cost and accessibility of these systems remain significant concerns, especially for elderly individuals with limited financial resources or technical expertize.

### 2.4 Research Gaps

Despite advancements in elderly monitoring systems and IoT technology, several research gaps remain unaddressed, which limit the effectiveness, scalability, and accessibility of existing solutions. These challenges highlight the need for further research and innovation in this field. One significant gap lies in the interoperability and standardization of various IoT devices and platforms used for elderly monitoring. The absence of standardized protocols and communication interfaces often leads to compatibility issues and isolated data systems, preventing seamless integration and data exchange across different platforms [31]. To overcome this barrier, there is a need to develop common standards and protocols that facilitate smooth interoperability among diverse devices and platforms.

Another critical gap involves privacy and security concerns associated with IoT‐based elderly monitoring systems. These systems handle sensitive health data, making it essential to ensure privacy, confidentiality, and robust security measures. However, many existing solutions lack adequate security mechanisms, exposing elderly users to risks such as data breaches and unauthorized access [27]. Future research must focus on improving security features in IoT platforms by integrating encryption, authentication, and access control mechanisms to protect sensitive information effectively.

This study seeks to address these research gaps by proposing an IoT‐based elderly monitoring system utilizing Firebase cloud computing. By leveraging Firebase's real‐time database, authentication, and security features, the proposed system enhances interoperability, scalability, and data security. Additionally, the study explores innovative approaches to integrating wearable devices, ambient sensors, and cloud services, aiming to provide comprehensive monitoring and timely intervention for elderly individuals. Through empirical evaluation and validation, this research contributes to advancing IoT‐based elderly care solutions and aims to bridge the existing gaps in the field.

## 3 Methodology

### 3.1 System Architecture

**Figure 1:**
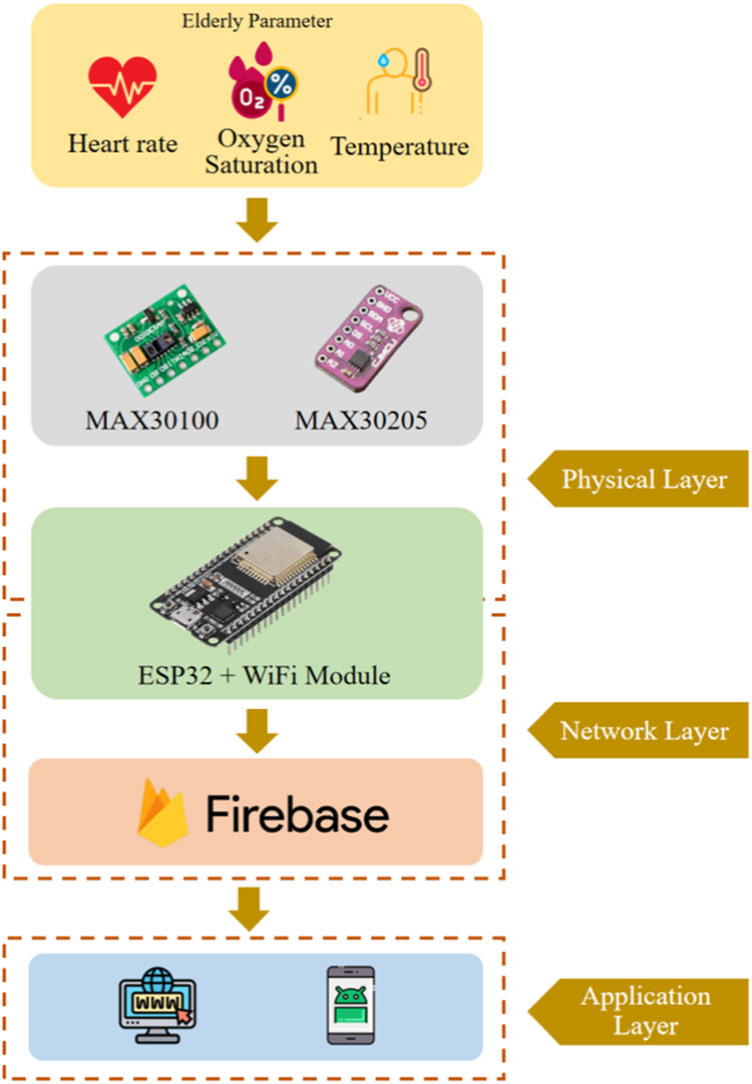
System architecture of elderly monitoring system.

The proposed system architecture is represented by Figure 1 where there are three main layers which refer to IoT construction which are divided into physical layer, network layer, and application layer. In the physical layer there is a physical sensing component in the form of a wearable sensor which can detect three parameters, namely, heart‐rate, oxygen saturation (SPO2), and temperature of human body. The sensor is of the MAX30100 type which employs to detect heart‐rate and oxygen saturation. Meanwhile, the MAX30205 is used to detect human body temperature. The ESP32 microcontroller is also integrated with the physical layer where data detected by physical sensing is then transmitted to the microcontroller for further processing. The ESP32 is equipped with a WiFi module so it can connect to the internet. At the network layer there is integration between ESP32 and the Firebase cloud system as data storage. ESP32 employs the message queuing telemetry transport (MQTT) in transmitting data into cloud platforms. The Firebase system receives this data and then stores the data in the cloud system to be accessed in real‐time by the user. In the application layer is a communication layer between user and system where real‐time data can be accessed via website or mobile‐based platforms. The implementation of machine learning is in the application layer where the system performs prediction tasks based on real‐time conditions including heart‐rate, oxygen saturation, and body temperature of the observed user. In this study, the machine learning technology employs supervised learning with a model mechanism that has been deployed in the application layer that classifies user conditions based on two class labels consisting of “stable” and “not stable.” Through the system that is built, it provides convenience for users not only to be able to find out the conditions based on the three parameters, but can also provide recommendations for stability conditions. Therefore, the proposed system could provide convenience to the user, low‐cost technology, and precision.

### 3.2 Hardware and Software Components

#### 3.2.1 Microcontroller ESP32

**Figure 2:**
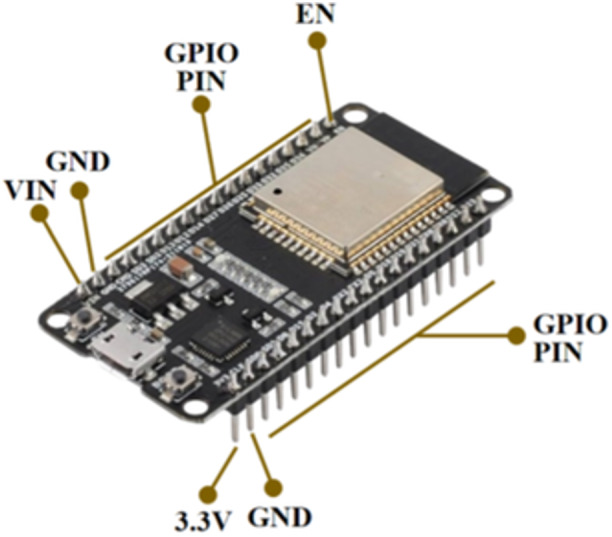
Microcontroller ESP32.

ESP32 is a microcontroller that supports IoT systems which is integrated with a built‐in Bluetooth and WiFi module. There are 36 pinouts consisting of EN, ground, VIN, 3.3 V, and GPIO pins. ESP32 employs Tensilica Xtensa as the microprocessor chip which has high speed performance in data processing. There is several memory integrated in the ESP32 which consists of ROM capacity of 448 kilo‐Bytes and SRAM capacity of 520 kilo‐Bytes. In addition, there are two memories used in sleep mode: RTC fast SRAM and RTC slow SRAM with the capacity of 8 kilo‐Bytes for each SRAM. The security standard of the system is IEEE 802.11 which can support security features, including WAPI, WFA, and WPA/WPA2. Figure 2 represents the detailed pinout specification of ESP32.

#### 3.2.2 MAX30100

**Figure 3:**
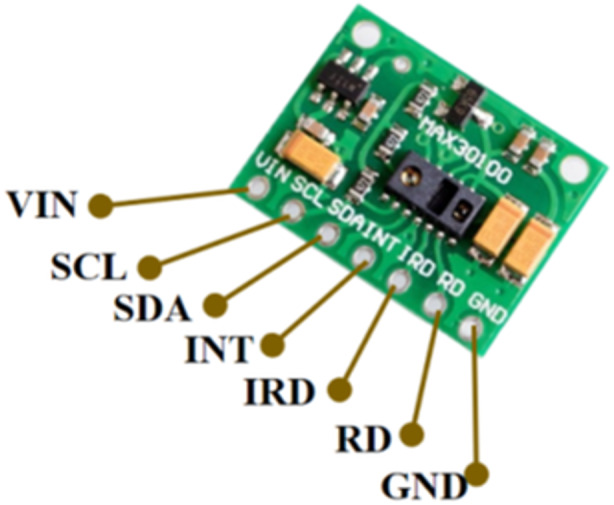
MAX30100 sensor.

MAX30100 is a wearable sensor which has the function of detecting heart‐rate and oxygen saturation in the human body as depicted in Figure 3. Has a similar working principle to an oximeter in reading oxygen saturation. Where there is a photodiode that detects oxygen saturation through skin touch. There are seven pins consisting of VIN, SCL, SDA, INT, IRD, RD, and ground. VIN functions as a voltage source where the optimal power supply range is between 3.3 V and 5 V. SCL is an I2C clock line that is connected to the microcontroller device and SDA is used to situate the I2C data. INT as a connector for detecting the pulse, IRD is used to indicate infrared LED whether detect the heart‐rate or oxygen saturation.

#### 3.2.3 MAX30205

**Figure 4:**
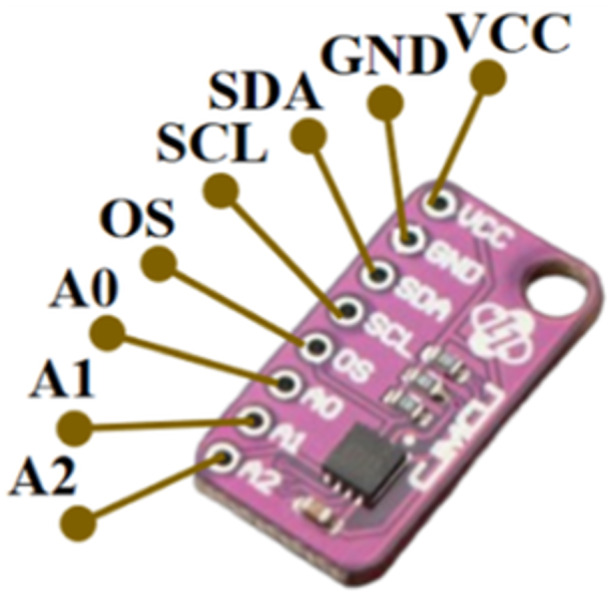
MAX30205 sensor.

The human body temperature can be measured by MAX30205 sensor, which can detect human temperature range up to 50°C and has an accuracy level of up to 0.1°C. The sensor can work on a supply voltage range between 2.7 V and 3.3 V and optimal current is 600 μA. There are eight pinouts consisting of VCC, GND, SDA, SCL, OS, A0, A1, A2, as depicted in Figure 4. VCC functions as a power source pin and GND as ground connector. SDA is used I2C data connector and SCL for I2C clock. A0, A1, and A2 are used for connecting into analog connectors. In this study MAX30205 will be employed as the wearable sensor to detect precisely the elderly people so as to provide the accuracy and valid data, also provide a low‐cost system to the user.

#### 3.2.4 Firebase Cloud System

**Figure 5:**
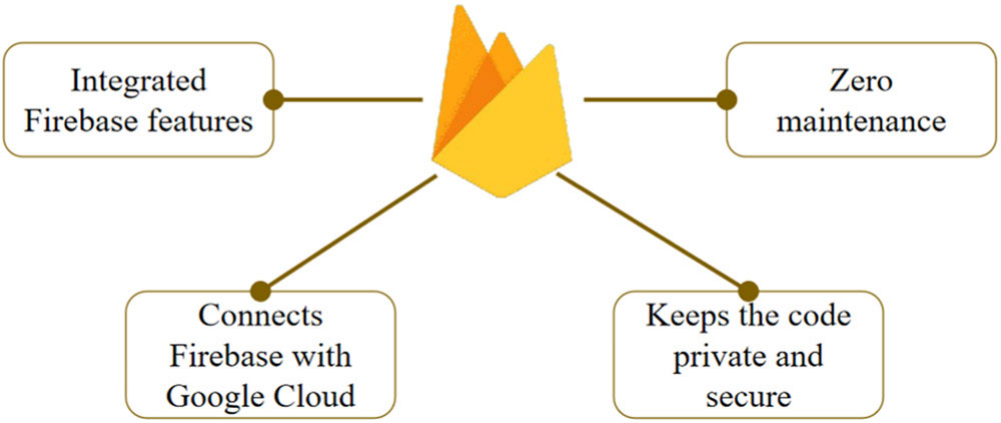
Firebase advantages.

Firebase is a cloud‐hosted database provided by Google, Inc Firebase can support various protocols including HTTP and MQTT. With this flexible interconnection capability, Firebase supports IoT‐based systems that require real‐time data access. Technically, data transmitted by IoT devices will be stored as JSON which is then accessed by users in real‐time. By enabling the secure access directly to the database from client‐side code, Firebase can provide complex and collaborative system applications. The end user which is on the application layer of the IoT architecture will have a responsive experience since data is stored locally and real‐time events continue to occur even when the device is offline. The real‐time data will be synchronized between local data change remotely when the device is offline and auto merging when the device is going to be online. Figure 5 represents the advantages of Firebase cloud system.

### 3.3 Data Collection and Transmission

**Figure 6:**
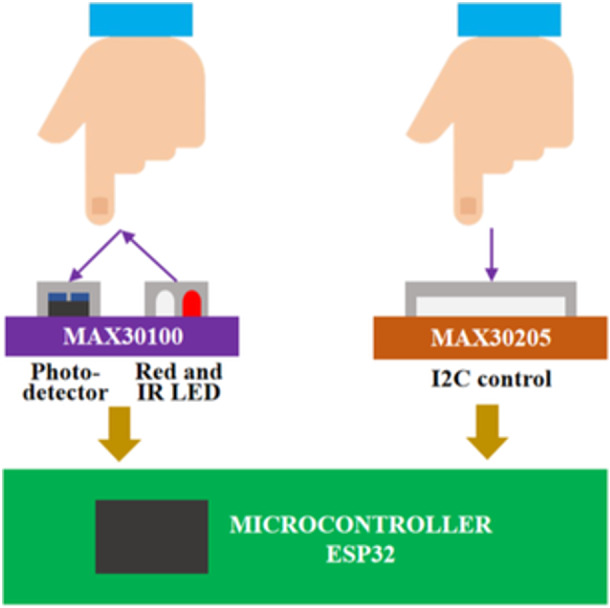
Wearable physical sensing data processing.

Wearable physical sensing on the MAX30100 and MAX30205 sensors as depicted in Figure 6 captures data through human body touch to obtain heart‐rate, oxygen saturation, and body temperature data. The LEDs have wavelengths of 660 nm and 880 nm, respectively. The MAX30100 works by shining both lights on the finger or earlobe and detecting the amount of reflected light with a photo‐detector. This method of detecting pulses using light is known as photoplethysmography. The MAX30100 works in two parts: heart‐rate measurement and oxygen saturation. The saturated oxygen in arterial blood has the property of absorbing infrared radiation. The higher the hemoglobin level, the more IR light is absorbed.

MAX30205 temperature sensor monitors temperature precisely and generates an over‐temperature alarm output. A high resolution, sigma‐delta, analog‐to‐digital converter (ADC) is used in this device to transform temperature measurements to digital form. An I2C‐compatible two‐wire serial interface enables access to the conversion results. The MAX30205 temperature sensor measures temperature accurately and outputs an over‐temperature alarm, interrupt, or shutdown. MAX30205 uses a high‐resolution sigma‐delta analog‐to‐digital converter (ADC) to convert temperature readings to digital form. Communication takes place over a 2‐wire serial interface that is I2C compliant. The I2C serial interface supports standard write byte, read byte, send byte, and receive byte commands for reading temperature data and configuring the open‐drain over temperature shutdown output behavior.

### 3.4 Data Communication and Analysis

**Figure 7:**
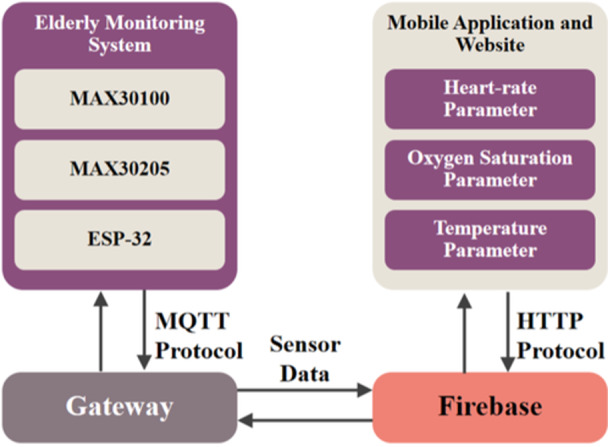
Device protocol communication.

The system protocols used in IoT‐based elderly monitoring system devices are MQTT and HTTP as depicted in Figure 7. The sensor device that has detected the three parameters and is processed by the microcontroller then transmits data via the built‐in WiFi module contained in the ESP32. The protocol mechanism used is MQTT. MQTT is a messaging protocol which is commonly used in IoT devices. MQTT enables resource‐constrained devices to send sensor data to a gateway which acts as a translator, then processes and stores the data with Firebase functionalities such as Cloud Functions or Realtime Database. The gateway system sends the data including heart‐rate, oxygen saturation, and temperature parameters to the Firebase cloud. Then, the data which has been stored to Firebase is accessed by mobile applications and websites using HTTP protocol. Users can see the current condition of the elderly people in the three parameters.

**Figure 8:**
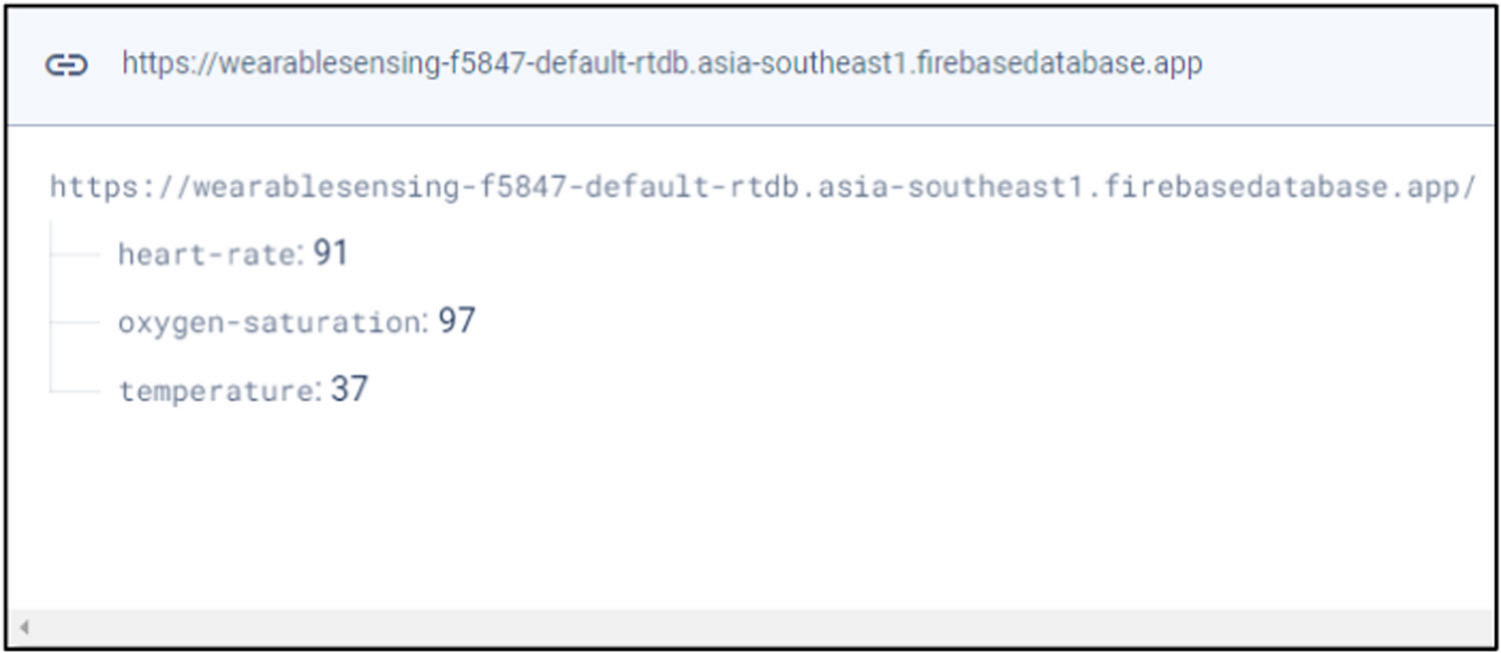
Sensing data from firebase.

Figure 8 depicts the realtime data in Firebase cloud system. Firebase provides Realtime Database and designed for real‐time updates. Whenever current sensor data arrives, Firebase sends it to linked devices via persistent WebSocket connections. This enables clients to examine the health vitals virtually immediately on mobile application and website.

### 3.5 System Implementation

**Figure 9:**
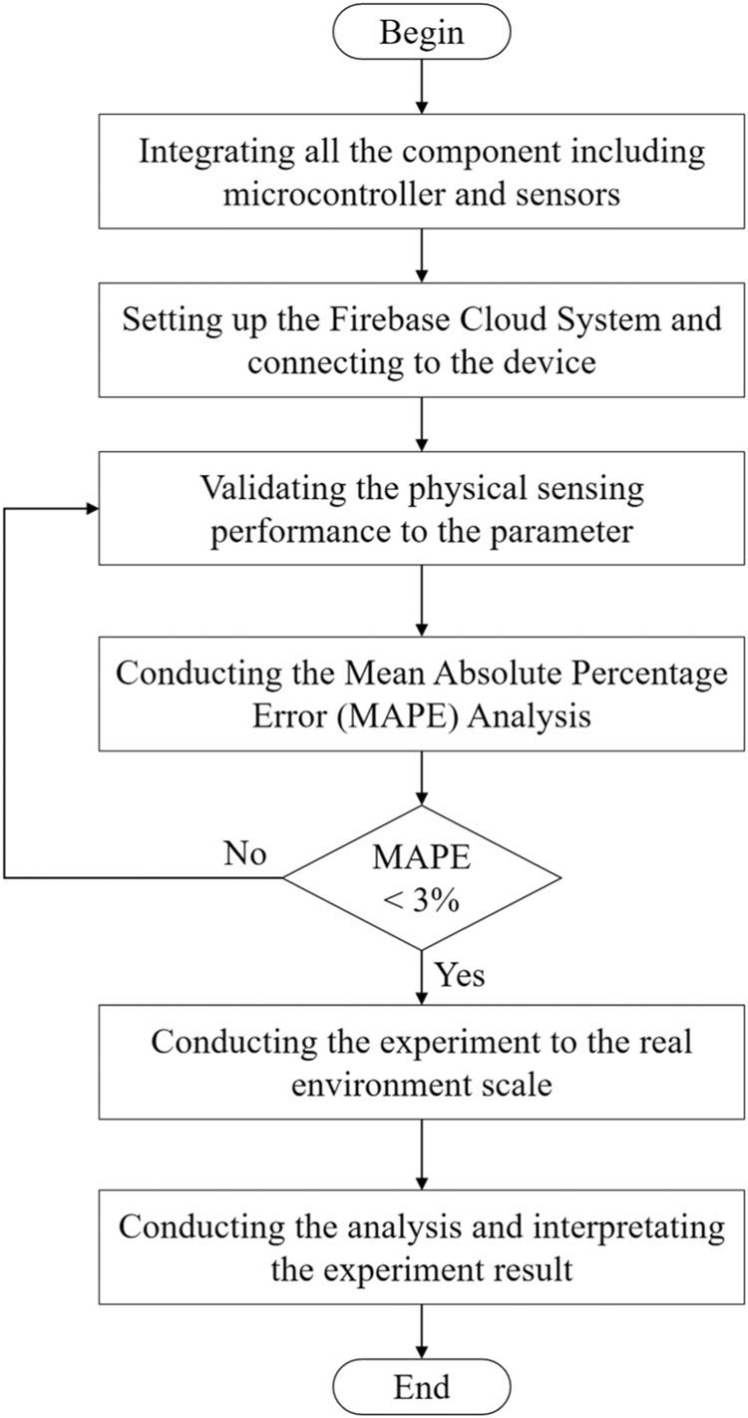
Experimental flowchart.

The experimental flowchart is represented by Figure 9 where the initial stage in the study is integrating all the components including MAX30100, MAX30205, ESP32 microcontroller, and other related components to the elderly monitoring system. The next stage is configuring the Firebase system by constructing the real‐time database, developing the initial key and values of heart‐rate, oxygen saturation, and human body temperature. Then, the Firebase is integrated to the IoT device and performing the connectivity testing. After all the system is integrated between hardware and software components, then validating the physical sensing performance of IoT device to the three parameters and comparing the results to the measurement tools such as digital oximeter and digital thermometer. At this stage involved the participants with the age range between 50 and 60 years. Several analyses are involved to ensure the device is functioning properly including mean absolute percentage error (MAPE) and accuracy level. The formulation of MAPE refers to Equation (1) as follow below:

(1) $$\mathrm{MAPE}=\frac{1}{n}\sum_{k=1}^{n}\left|\frac{A_{k}-P_{k}}{A_{k}}\right|.$$

Where *n* indicates the summation iteration number, *A*_*k*_ and *P*_*k*_ represent the actual and predicted value, respectively. In the study, the MAPE threshold is below 3% which means if above the threshold it will iterate to the previous stage, and if below the threshold it will continue to the experiment stage in the real‐world data. At this stage participants will be involved with several conditions to see how the IoT responds to the current participant condition, IoT device time‐response, and the user feedback

As a reference parameter in conducting experiments in this study, Table 1 provides the standards of heart‐rate, oxygen saturation, and temperature for the elderly people in the age range between 50 and 90 [15, 20]. The units used in heart rate is beats per minute or BPM where each age has a different range, for example at age 50 the range is 85 to 119 BPM. Meanwhile, in the age range of 60 is between 80 and 112 BPM. In oxygen saturation parameter has the same range between ages of 50 and 90, that is 95% to 100%. Meanwhile, temperature parameters at ages age of 50 and 60 has the range between 36.4°C and 37.6°C. At the age of 70 to 90 has the range between 35.8°C and 36.9°C

**Table 1:** Normal condition of each parameter.

Parameter	Age				
	50	60	70	80	90
Heart‐rate (beat per minute [BPM])	85–119	80–112	75–105	70–98	65–91
Oxygen saturation (%)	95–100	95–100	95–100	95–100	95–100
Temperature (°C)	36.4–37.6	36.4–37.6	35.8–36.9	35.8–36.9	35.8–36.9

Table 2 represents the scenario when the device is detecting the real‐world data where there are four scenarios including sleeping, walking, sitting, and cardio exercising. The purpose of the experiment is to see how responsive the device is in detecting various human conditions from resting positions to doing cardio activities such as cardio exercise.

**Table 2:** Experiment scenario in real‐world application.

ID	Scenario	Description
1	Sleeping	Participants were lying down and resting by closing their eyes in a sleeping position. Data collection was carried out 30 min after the participant lay on the bed
2	Walking	Participants did light walking for 30 min. After that, experimental data was collected
3	Sitting	Participants rested in a sitting position and occasionally watched movies and chatted. Data collection was carried out after 30 min of participants sitting and resting
4	Cardio Exercising	Participants did brisk walking and running for 15 min. After that, experimental data was collected

## 4 Results and Discussion

### 4.1 System Performance Validation

Device validation was carried out in three stages by measuring both monitoring devices and measurement tools. The first stage is carried out by measuring heart‐rate parameters, while the second and third stages are carried out by measuring oxygen saturation and human body temperature, respectively. In the experiment melibatkan enam partisipan dengan rentang usia 50 to 65 years. Table 3 merepresentasikan participants's information dimana terdapat dua partisipan yang memiliki chronic disease and health condition “not‐stable” dengan ID P‐004 and P‐006 with the age of 63 and 65 years, respectively. Data was collected every second during 10 min with the participants in a resting of sitting position. Before carrying out the device validation test, an interview process was carried out to see the health history of the participant's condition and participant assign the letter of consent that willing to be involved in the experiment.

**Table 3:** Participants' information in system performance validation experiment.

Participant's ID	Age	Chronic disease	Smoking	Condition
P‐001	50	No	Rarely	Normal
P‐002	55	No	No	Normal
P‐003	60	No	No	Normal
P‐004	63	Yes	No	Not stable
P‐005	65	No	Rarely	Normal
P‐006	65	Yes	No	Not stable

From the total duration of 10 min, resulting in 600 data records representing the participant's heart‐rate, oxygen saturation and temperature. The resulting data is time‐series data taken every second. Table 4 shows the results of the experiment by collecting data via monitoring devices and measurement tools for the six participants. Meanwhile, Tables 5 and 6 are data retrieval for oxygen saturation and temperature, respectively. #Dev denotes as the result taken by the monitoring device and #Tls denotes as the result based on measurement tools. Each participant which indicated by P‐001 to P‐006 have the result of #Dev and #Tls.

**Table 4:** Experiment result of participants' heart‐rate.

Time	ID	P‐001		P‐002		P‐003		P‐004		P‐005		P‐006	
		#Dev	#Tls	#Dev	#Tls	#Dev	#Tls	#Dev	#Tls	#Dev	#Tls	#Dev	#Tls
00:00:01	1	86.2	86.4	84.3	84.5	82.2	82.1	92.5	92.7	88.9	89.3	98.3	98.7
00:00:02	2	86.1	86.4	83.1	83.2	84.3	84.5	92.1	92.3	88.5	88.9	98.1	98.3
00:00:03	3	86.2	86.4	85.6	85.7	82.1	82.2	92.6	92.8	88.2	88.6	97.6	97.7
00:00:04	4	85.4	85.8	86.4	86.5	87.3	87.5	89.3	89.5	89.3	89.5	98.3	98.5
00:00:05	5	86.5	86.9	86.3	86.3	88.2	88.3	90.1	90.2	88.8	89.0	98.0	98.4
00:00:06	6	84.8	84.2	84.8	84.8	92.3	92.6	91.5	91.6	87.2	87.4	97.6	98.0
00:00:07	7	85.2	85.6	80.2	80.2	89.1	89.2	90.8	91.0	86.1	86.3	97.5	97.6
00:00:08	8	88.3	88.6	84.8	84.8	86.3	86.4	92.5	92.7	88.3	88.5	98.4	98.6
00:00:09	9	87.2	87.4	82.6	82.6	89.6	89.6	93.2	93.2	88.2	88.4	98.1	98.5
00:00:10	10	87.3	87.5	79.9	79.9	80.1	80.4	93.0	93.2	88.5	88.6	97.5	97.6
…	…	…	…	…	…	…	…	…	…	…	…	…	…
00:05:00	600	91.7	92.3	80.2	80.3	79.1	79.2	87.2	87.4	89.1	89.2	98.2	98.4

**Table 5:** Experiment result of participants' oxygen saturation.

Time	ID	P‐001		P‐002		P‐003		P‐004		P‐005		P‐006	
		#Dev	#Tls	#Dev	#Tls	#Dev	#Tls	#Dev	#Tls	#Dev	#Tls	#Dev	#Tls
00:00:01	1	95.8	95.9	99.1	99.4	99.2	99.7	97.2	97.3	98.2	98.3	97.0	97.4
00:00:02	2	99.1	90.4	97.4	97.1	98.8	98.1	97.8	98.2	98.0	98.4	97.3	97.7
00:00:03	3	97.4	97.1	89.6	98.2	95.4	95.3	97.5	97.6	98.3	98.4	97.1	97.5
00:00:04	4	99.6	99.2	92.1	97.7	98.7	98.8	98.2	98.4	97.2	97.4	97.4	97.8
00:00:05	5	92.1	97.7	95.5	95.1	99.5	99.6	97.1	97.2	98.5	98.6	97.2	97.4
00:00:06	6	95.5	95.1	99.1	99.4	99.7	99.9	98.2	98.6	97.2	97.6	97.5	97.9
00:00:07	7	99.1	99.4	95.8	95.9	99.5	99.7	98.1	98.5	97.9	98.3	97.9	98.1
00:00:08	8	95.8	95.9	99.1	90.4	92.7	92.8	98.4	98.6	98.1	98.3	98.2	98.6
00:00:09	9	99.1	90.4	97.4	97.1	99.7	99.8	97.2	97.4	98.5	98.7	98.2	98.4
00:00:10	10	97.4	97.1	89.6	98.2	99.5	99.7	97.3	97.7	98.7	99.1	97.7	98.1
…	…	…	…	…	…	…	…	…	…	…	…	…	…
00:05:00	600	99.5	99.6	97.4	97.1	99.5	99.6	97.2	97.6	97.1	97.5	97.3	97.5

**Table 6:** Experiment result of participants' temperature.

Time	ID	P‐001		P‐002		P‐003		P‐004		P‐005		P‐006	
		#Dev	#Tls	#Dev	#Tls	#Dev	#Tls	#Dev	#Tls	#Dev	#Tls	#Dev	#Tls
00:00:01	1	36.4	36.4	36.8	36.8	37.5	37.6	36.8	36.8	37.3	37.3	36.6	36.6
00:00:02	2	36.7	36.8	36.5	36.4	36.1	36.4	36.8	36.7	37.2	37.4	36.5	36.6
00:00:03	3	36.7	36.8	36.7	36.8	36.7	36.8	36.9	36.9	37.3	37.5	36.5	36.6
00:00:04	4	36.1	36.1	36.6	36.4	36.2	36.2	37.0	37.2	37.0	37.0	36.8	36.7
00:00:05	5	36.7	36.8	36.7	36.8	36.4	36.6	37.0	37.2	36.8	37.0	36.7	36.7
00:00:06	6	36.1	36.4	36.2	36.2	36.7	36.8	37.1	37.1	36.9	37.0	36.8	36.7
00:00:07	7	36.7	36.8	36.4	36.6	36.2	36.2	36.8	36.7	37.1	37.0	37.0	36.8
00:00:08	8	36.2	36.2	36.7	36.8	36.2	36.2	36.9	37.2	36.9	36.9	36.9	36.9
00:00:09	9	36.4	36.6	36.7	36.8	36.4	36.6	36.6	36.6	36.8	36.9	37.0	36.9
00:00:10	10	36.2	36.2	36.7	36.8	36.7	36.8	36.7	36.8	37.0	36.9	37.0	36.9
…	…	…	…	…	…	…	…	…	…	…	…	…	…
00:05:00	600	37.1	37.2	36.2	36.3	36.4	36.4	37.0	36.9	37.2	37.2	36.7	36.8

To facilitate the subsequent analysis process, an average value was carried out for the results from participants which were categorized based on parameters and measurement instruments including IoT devices and tools as represented in Table 7 where we can see the average result of each parameter. For example, the first data collection carried out at time 00:00:01 resulted in the device's average value for the heart‐rate parameter being 84.35 BPM, while the average produced by the measurement tools was 84.33 BPM. Likewise with the oxygen saturation result, where the average for IoT devices is 98.23% and measurement tools is 98.54%. Meanwhile, in body temperature results, the average device and measurement tools are indicated by 36.72°C and 36.84°C

**Table 7:** Average result of heart‐rate, oxygen saturation, and temperature parameter.

Time	ID	Heart‐rate (BPM)		Oxygen saturation (%)		Body temperature (°C)	
		Device	Tools	Device	Tools	Device	Tools
00:00:01	1	84.35	84.33	98.26	98.55	36.69	36.86
00:00:02	2	80.20	80.38	98.53	95.31	36.27	36.55
00:00:03	3	78.95	79.23	94.36	97.11	36.67	36.82
00:00:04	4	87.68	87.91	96.98	98.77	36.19	36.18
00:00:05	5	87.12	87.35	95.61	97.38	36.66	36.68
00:00:06	6	84.72	84.94	98.01	98.04	36.39	36.42
00:00:07	7	87.82	88.04	98.04	98.24	36.49	36.48
00:00:08	8	85.78	85.89	95.78	92.94	36.43	36.43
00:00:09	9	84.25	84.32	98.64	95.68	36.56	36.7
00:00:10	10	80.22	80.39	95.41	98.24	36.59	36.63
…	…	…	…				
00:05:00	600	83.89	84.12	98.91	98.9	36.63	36.58

**Figure 10:**
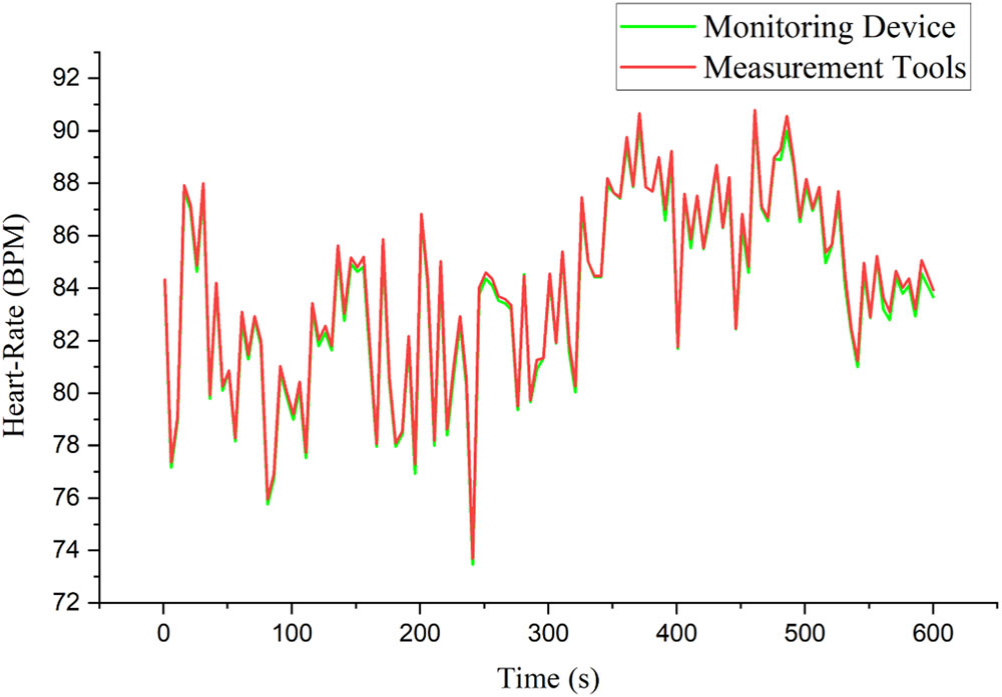
Graphic of heart‐rate parameter result.

Figure 10 represents the average graph of the heart‐rate parameter result where the two measurement results of both IoT devices and measurement tools do not appear to have a significant difference. Axis x indicates the time range in units of seconds (s) for 600 s, axis y indicates the heart‐rate result in BPM units. During the data collection range at time 1 to 600, the increasing and decreasing fluctuations of the two devices have the same tendency even though there is a difference in results between the range 0.1 to 0.3.

**Figure 11:**
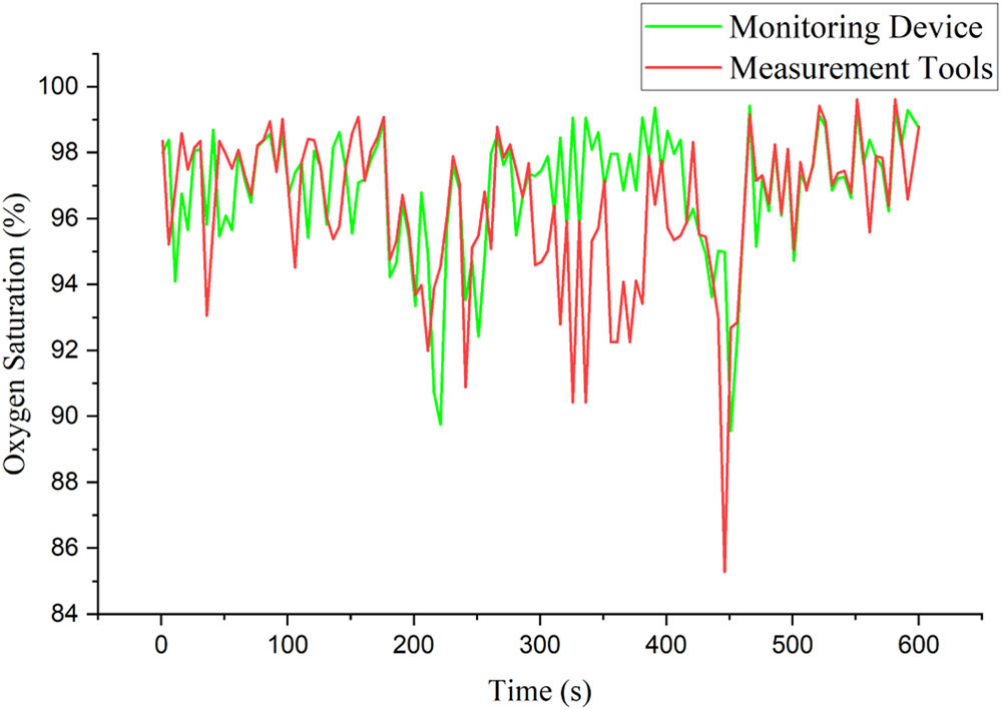
Graphic of oxygen saturation parameter result.

The oxygen saturation graph is represented by Figure 11, y axis is oxygen saturation with unit is percentage (%). If observed in more detail, there is a significant gap in the 340 and 380 time range where the average produced by the device which is indicated by the green line is higher than the measurement tool which is indicated by the red line. However, in the time range of 480 to 550 s, the tendency of measurement results between IoT devices and measurement tools is the same.

**Figure 12:**
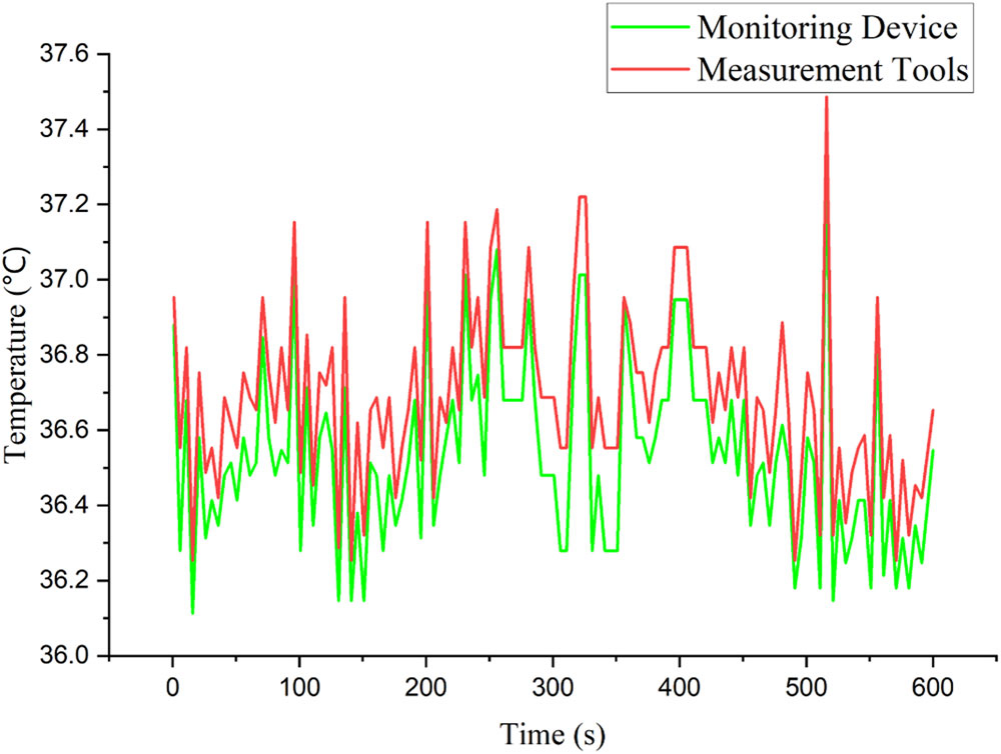
Graphic of temperature parameter result.

Meanwhile, Figure 12 indicates the average result graph of temperature parameters. The average measurement range during data collection from 1 to 400 s is 36°C to 37.5°C. In general, the increasing and decreasing fluctuations resulting from both devices have the same tendency. However, if you look in more detail at the graph, there is a fairly wide gap between the two devices, especially at time 300 to 360. And then the gap between the two devices narrows again at time seconds from 500 to 600.

The next stage is to carry out a MAPE analysis which is part of the device validation. In this study the threshold MAPE is less than 3% as described in Figure 8. Table 8 represents the MAPE analysis of the three parameters including heart‐rate, oxygen saturation, and body temperature. From each of these parameters there are measurement instrument results in the form of devices and tools. The difference in the results of the two instruments is hereinafter referred to as the “gap.” In the heart‐rate parameter, the resulting gap is 1.42 BPM, which means the difference in the heart‐rate measurement results produced by the IoT device and the measurement tools. Likewise, the gaps in oxygen saturation and body temperature are 0.55% and 0.16°C, respectively. The gap is the basis for calculating the percentage error for each parameter by calculating the division between the gap and the measurement results of the measurement tools as a measurement reference. For example, the resulting heart‐rate gap is 1.42 BPM, then divided by the measurement tool measurement result of 85.79 BPM, then multiplied by 100%, then the percentage error of the heart‐rate measurement results is 1.68%. The same method for calculating percentage error was also implemented for the oxygen saturation and body temperature parameters which resulted in the percentage error of 0.57% and 0.44%, respectively. The results of the three percentage error parameters are then averaged and produce a value of MAPE of 0.90%. This means that the results of the MAPE are lower than the threshold determined in this study. To find the accuracy value, do a 100% calculation which is reduced by the value of MAPE, which produces an accuracy percentage of 99.10%.

**Table 8:** Mean absolute percentage error (MAPE) analysis.

Parameter	Measurement instrument	Value	Percentage error
Heart‐rate (BPM)	Device	84.37	1.68%
	Tools	85.79	
	Gap	1.42	
Oxygen saturation (%)	Device	96.85	0.57%
	Tools	96.30	
	Gap	0.55	
Body temperature (°C)	Device	36.53	0.44%
	Tools	36.69	
	Gap	0.16	
**Mean absolute percentage error (MAPE)**			**0.90%**
**Accuracy (100% – MAPE)**			**99.10%**

### 4.2 The Implementation of Machine Learning

In addition to using IoT and cloud computing technology, the developed device implements machine learning technology where the device can predict the patient's condition whether it has a tendency toward “stable” or “not stable” conditions. The implementation of machine learning modeling used in this study is supervised learning with a classification model. There are several stages in the development of a machine learning model including dataset development, exploration data analysis, data transformation, model training, model evaluation, and deployment.

#### 4.2.1 Developing Dataset

As a database for machine learning to have knowledge of how to perform classification tasks, a dataset that is relevant to the domain being discussed is needed. In this study, the dataset was developed through data obtained from participants and data obtained from the public repository kaggle. com. The dataset used is tabular data consisting of features and class labels. Features are parameters used as descriptions of each data record, consisting of heart‐rate, oxygen saturation, and body temperature. While the class label represents a classification consisting of the labels “stable” and “not stable” which are based on the normal conditions of heart‐rate [31], oxygen saturation [31], and body temperature [31] as represented in Tables 9, 10, 11, respectively.

**Table 9:** Heart‐rate normal condition based on age.

Age	Range of heart‐rate (BPM)
20	100–170
30	95–162
35	93–157
40	90–153
45	88–149
50	85–145
55	83–140
60	80–136
65	78–132
70	75–128

**Table 10:** Oxygen saturation in human body.

Criteria of oxygen saturation	Percentage rate
Low	Less than 91%
Medium	91%–98%
High	99%–100%

**Table 11:** Temperature of human body based on age.

Age	Degree celcius
11–65	36.4–37.6
66–96	35.8–36.9

Furthermore, based on the three tables above, a dataset was developed with 4500 data records consisting of the class labels “stable” and “not stable.” For data with the class label “stable” refers to the normal conditions contained in Tables 9, 10, 11. While for conditions that do not meet the standard conditions of the three Tables, they will be included in the class label “not stable.” in this study, the division of data for the class labels “stable” and “not stable” was carried out evenly with 2250 data records each. Table 12 represents the dataset used in this study.

**Table 12:** Dataset elderly health monitoring system.

ID	Heart‐rate	Oxygen saturation	Body temperature	Class
1	92	95	37	Stable
2	87	95	37	Stable
3	110	100	36	Stable
4	103	98	36	Stable
5	97	97	36	Stable
6	105	98	40	Not stable
7	92	90	39	Not stable
8	65	88	36	Not stable
9	86	95	37	Stable
10	113	98	36	Stable
…	…	…	…	…
4500	75	82	39	Not stable

To facilitate the model in recognizing data during model training, so as to produce good performance accuracy, data transformation is carried out on the three features including heart‐rate, oxygen saturation, and body temperature. The transformation process is carried out by employing numbers 0, 1, and 2 to represent low, normal, and high conditions, respectively. For example, in body temperature with ID = 6, it can be seen that the temperature is 40°C and this is a high temperature condition. So that the data is transformed into the number 2. Likewise with oxygen saturation at ID = 3 which shows 100% or in normal conditions, then the transformation is represented into the number 1. The class label also undergoes a transformation process, where the label “stable” is represented by the number 1 and “not stable” is represented by the number 0. Table 13 represents the results of the dataset transformation.

**Table 13:** Dataset transformation.

ID	Heart‐rate	Oxygen saturation	Body temperature	Class
1	1	1	1	1
2	1	1	1	1
3	1	1	1	1
4	1	1	1	1
5	1	1	1	1
6	1	1	2	0
7	1	0	2	0
8	0	0	1	0
9	1	1	1	1
10	1	1	1	1
…	…	…	…	…
4500	1	0	2	0

#### 4.2.2 Classification Model Training and Evaluation

The next step is to implement the supervised model on the dataset that has been developed. In this study, the dataset is divided into three data categories including training, validation, and testing data with the percentage of 70%, 10%, and 20%, respectively. Specifically, the division of the dataset can be seen in the following Table 14:

**Table 14:** Training, validation, and testing data.

Class	Training	Validation	Testing	Total data
**Stable**	1575	225	450	2250
**Not stable**	1575	225	450	2250
**Total**	**3150**	**450**	**900**	**4500**

There are five supervised models that will be compared to see which model has the most optimal performance, including Adaptive Boosting (AdaBoost), XGBoost, Decision Tree Classifier, Random Forest, and Bayesian Classifier. To measure the performance of the five models, evaluation indicators are used, consisting of accuracy, F1 score, precision, and recall. Accuracy shows the percentage of data that has a true value (including positive or negative data) to the total data. Precision represents the percentage of data that has a true value based on all positive predictions. Recall represents all true positive values divided by the sum of true positives and false negatives. While F1 score is the value of averaged precision and recall. In modeling, it is necessary to determine the initial parameter configuration consisting of epoch of 300, learning rate 0.001, sigmoid activation function, and optimizer Adam. While the device configuration in training the model is represented in Table 15.

**Table 15:** Device configuration.

Description	Specification
Software	Jupyter Notebook
Programming language	Python
Processor	Intel Core i7 4770 3.40 Giga‐Hertz
Random access memory (RAM)	8.00 Giga‐Byte
Operating system	Windows 11.0

Table 16 represents the experiment result of five training models with evaluation metrics including precision, recall, accuracy, and F1 score. Overall, the accuracy produced by the five models is more than 0.9 whereas the XGBoost model has the highest accuracy with a value of 0.973. The AdaBoost model has an accuracy of 0.006 lower than XGBoost with an accuracy value of 0.967. From the F1 score aspect, the five models indicate that the XGBoost model has the highest value, which produces an F1 score of 0.970. The other five models including AdaBoost, Decision Tree Classifier, Random Forest, and Bayesian Classifier have F1 scores of 0.946, 0.933, 0.929, 0.928, respectively. Overall, the four evaluation metrics indicate that XGBoost has the highest performance.

**Table 16:** Model performance result.

Model name	Precision	Recall	Accuracy	F1 score
Adaptive boosting (AdaBoost)	0.956	0.937	0.967	0.946
**XGBoost**	0.973	0.968	0.973	0.970
Decision tree classifier	0.941	0.925	0.931	0.933
Random forest	0.936	0.922	0.946	0.929
Bayesian classifier	0.925	0.931	0.931	0.928

**Figure 13:**
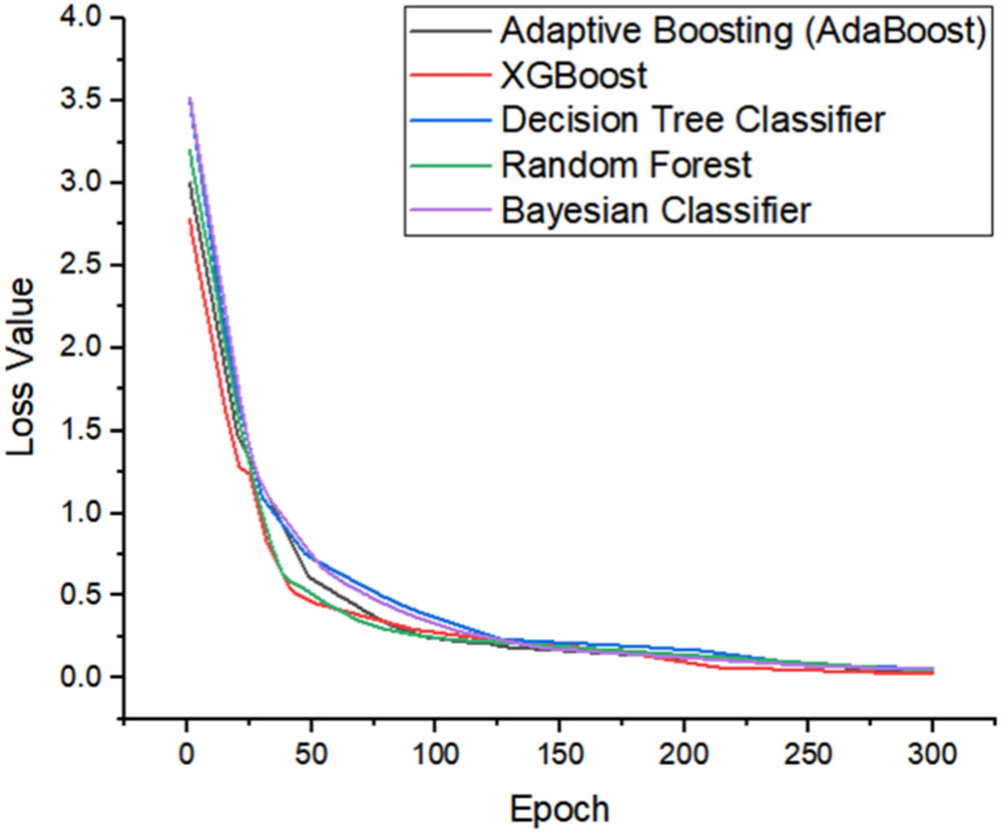
Training loss of the compared five model.

Loss value indicates the level of error generated by the model, in other words, how misclassification is conducted by the model. The lower the loss value, the better of model's performance. Figure 13 shows the loss value result on the five models during the training process in 300 epochs with the AdaBoost model represented by a black line color, XGBoost as a red line color, Decision Tree Classifier is blue line color, Random Forest is green line color, and Bayesian Classifier is purple line color. Overall, the loss value generated by the five models is below 0.1 where the highest loss value is in the Bayesian Classifier model with a value of 0.062. The lowest loss value is in the XGBoost model with a value of 0.025. Meanwhile, the AdaBoost, Decision Tree Classifier, and Random Forest models have a loss value of 0.033, 0.058, and 0.048, respectively. Overall, based on the result of metrics including precision, recall, accuracy, F1 Score, and loss value, XGBoost has the most optimal performance. Therefore, in this study, XGBoost will be selected as the modeling algorithm to conduct the classification task based on the parameters of heart‐rate, oxygen saturation, and body temperature and predict user condition whether in “stable” or “not stable” condition.

### 4.3 Real‐World Data Experiment

#### 4.3.1 User Interface Design

To facilitate interaction between the user and the system, an Android mobile‐based user interface was designed. User interface development using the Android Studio Integrated Development Environment (IDE) application with the Java programming language for logic content and the XML programming language for display design. In the mobile device implementation stage, minimum specifications are required so that the system can run optimally on Android‐based mobile devices. Table 17 is the minimum mobile device specifications.

**Table 17:** Mobile device minimum requirement.

Description	Specification
Basic operating system	Android
Android version	Lollipop (5.0)
Internal free storage space	1 Giga‐byte
Random access memory (RAM)	2.0 Giga‐byte
Processor clock	1.2 Giga‐hertz (quad‐core)
Connection	WiFi and minimum 4G connectivity

**Figure 14:**
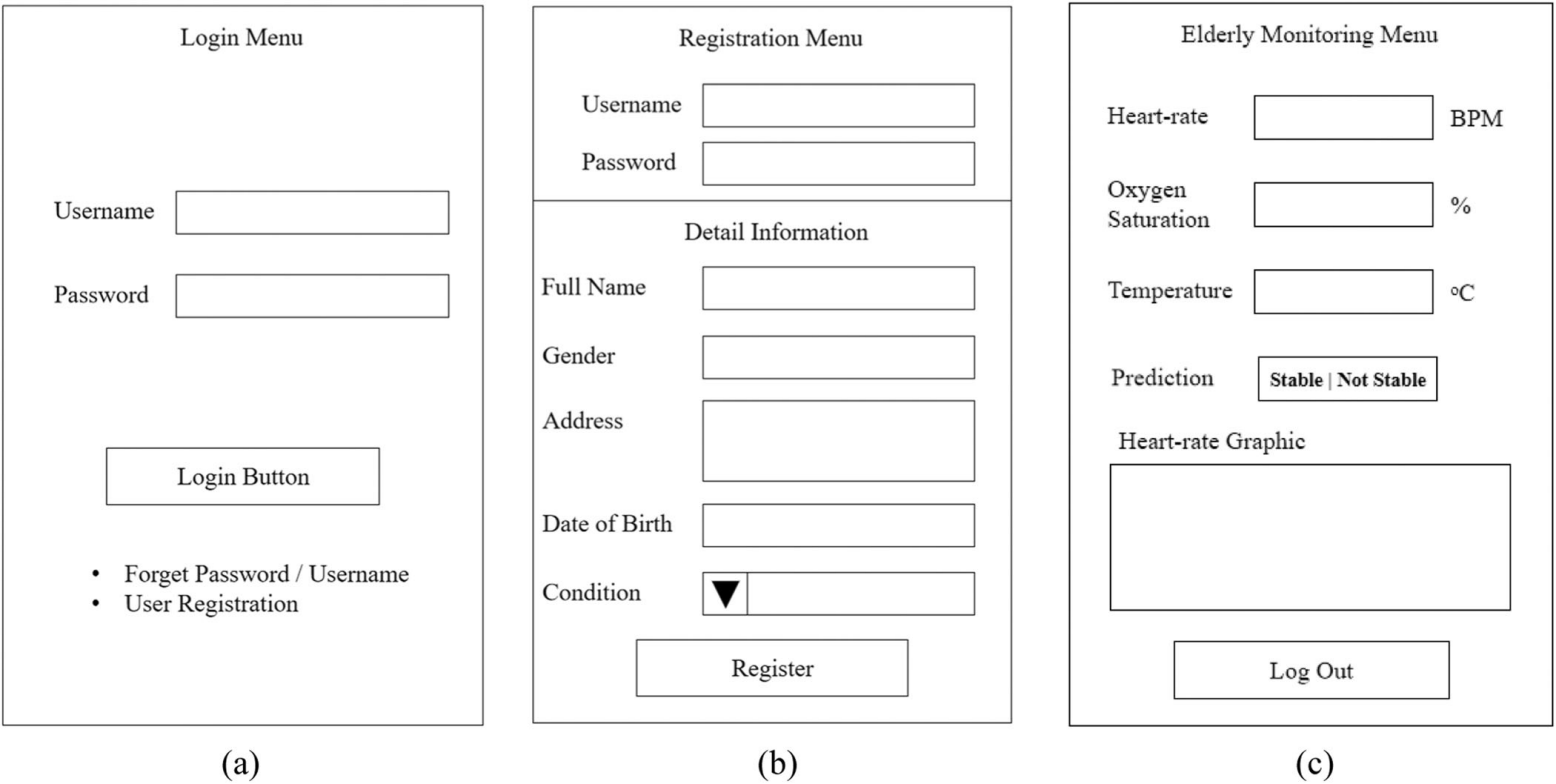
User interface design. (A) Login menu. (B) Registration menu. (C) Elderly monitoring menu.

Figure 14 is a user interface display design which has three main user interfaces which are divided into login menu, registration menu, and monitoring menu. In Figure 14A is a login menu where the system carries out the user authentication process, thus guaranteeing the confidentiality of user data. Each user must register an account as initial basic information from the user, as depicted in Figure 14B. There are data fields that must be input by the user including user full name, gender (male or female), address, date of birth which will automatically display the age of the user, as well as the current condition of the user whether optimal or less optimal. If the user chooses less optimal, then the reasons need to be stated which are related to the user's health condition. Figure 14C represents current condition of the user in parameters of heart‐rate (BPM), oxygen saturation (%), temperature (°C), and predicted classification of the model whether “stable” or “not stable.” As supporting data, there is a graph that shows the conditions taken every second. Graphic will update every 5 min.

**Figure 15:**
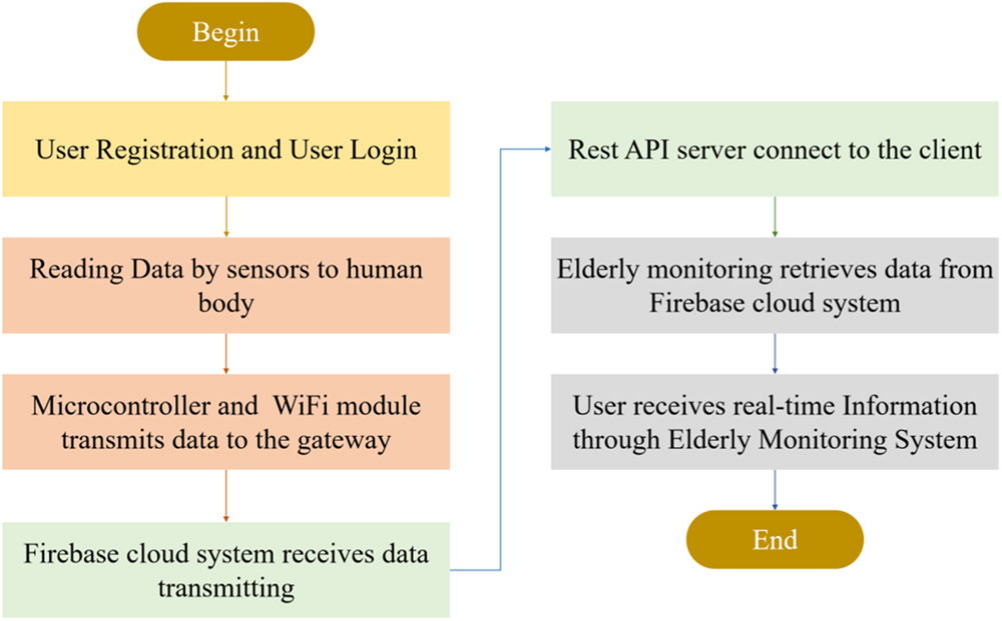
System flowchart.

Software and hardware integration is carried out to produce valid, smooth and integrated information output. The IoT architecture implemented in the study is able to accommodate the integration process through the proposed three layer architecture, including physical layer, network layer, and application. However, detailed steps need to be taken to ensure data integration runs smoothly and is standardized. Figure 15 is how the software and hardware integration process is represented in the system flowchart. The stage begins with user registration as initial validation of user data recorded in a confidential manner. Next is the process of reading sensors and transmitting data to the cloud gateway which is finally received by the Firebase cloud system. From the client side, through the rest API, client‐server connectivity occurs so that the elderly monitoring application retrieves data from the Firebase cloud system and the user can receive real‐time information through the application.

#### 4.3.2 Real‐World Practical Application

An Android mobile‐based practical application experiment was carried out to find out how the system performs in real‐world data. As described in Table 2, the real‐world practical application scenario refers to four participant conditions, including (1) sleeping; (2) walking; (3) sitting; and (4) cardio exercise using treadmill. An initial assessment was carried out to determine the characteristics of the participants, who were males aged 50 years. Health condition is good and has no history of chronic disease. Participants are involved in real‐world experiments for all experimental scenarios. The data collection process was carried out on different days and there was a 1 day gap between each data collection. This aims to ensure the validity of the data produced and that participants are in stable condition. During the data collection process, the participant's condition was stable and their health was normal. Table 18 is the initial participant data.

**Table 18:** Real‐world experiment participant.

Parameter	Description
Gender	Male
Age	50 years old
Smoking	No
Chronic disease	No
Hypertension	No
Alcoholic	No
General condition	Normal

**Figure 16:**
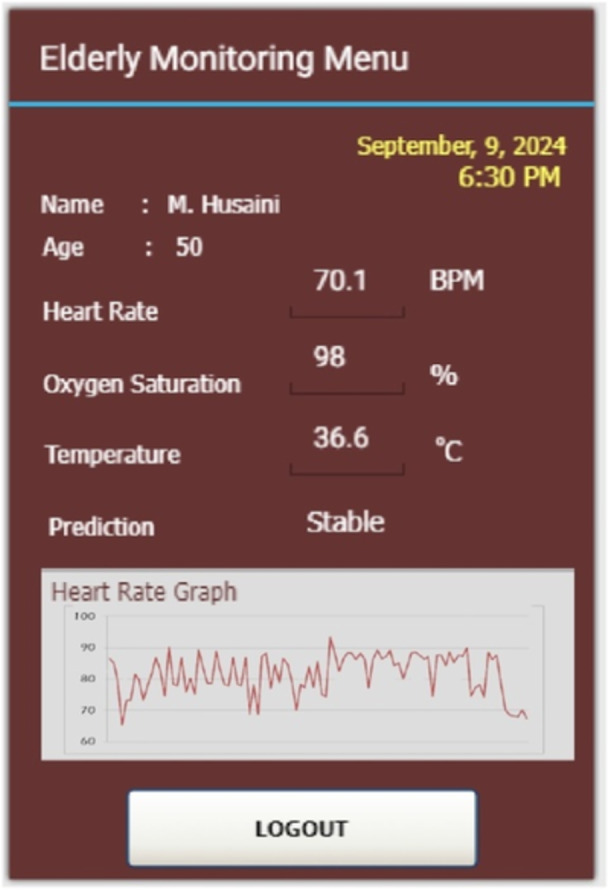
Real‐world experiment for sleeping condition.

Figure 16 is the result of a real‐world experiment for sleeping conditions. Referring to Table 2, where data collection was carried out after the participant had fallen asleep for 30 min. Measurements of the participants' initial condition before data collection were carried out for heart‐rate, oxygen saturation, and temperature parameters with values of 75.8 BPM, 97.5%, and 36.7°C, respectively. When sleeping, the heart‐rate decreases to 70.1 BPM. Meanwhile, the results of oxygen saturation and temperature measurements were 98% and 36.6°C, respectively. Meanwhile, the prediction condition of current data is “stable.”

**Figure 17:**
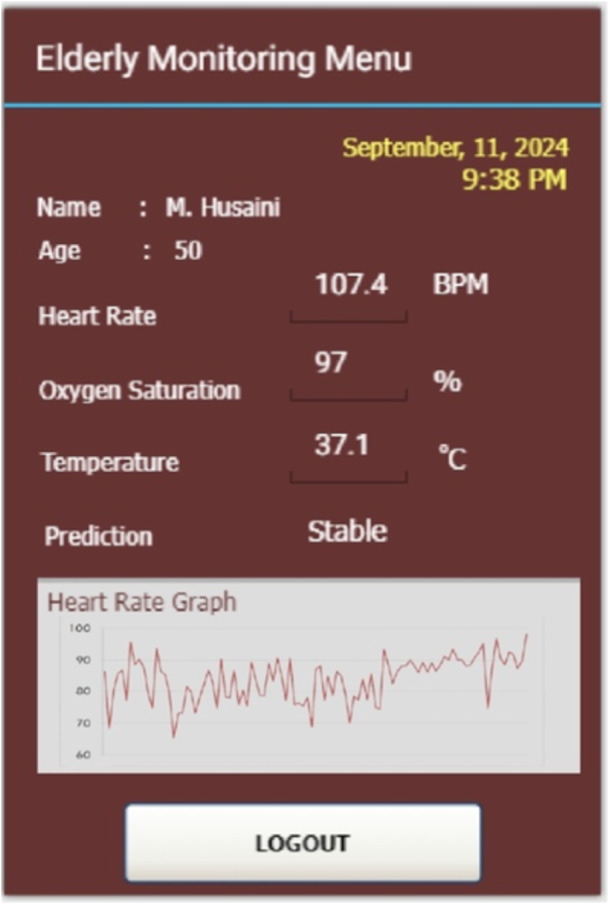
Real‐world experiment for walking condition.

In the experimental walking condition as depicted in Figure 17 found that heart‐rate, oxygen saturation, and temperature were 107.4 BPM, 97%, and 37.1°C, respectively. Compared to sleeping condition, the participant's parameter measurement results in the walking condition increased. This is because there is physical activity that triggers an increase in these parameters. These results are consistent with previous studies [30, 31], which indicate that physical activity can significantly increase vital body parameters, as the body requires more oxygen and energy during physical exertion. Monitoring these vital signs is particularly important in older adults, who are more susceptible to sudden physiological changes. Health monitoring systems based on IoT technology, as employed in this study, provide accurate, real‐time information about the physical condition of elderly individuals, enabling early detection of potential health issues and more timely interventions. Thus, the use of such monitoring technologies can help improve the quality of life and well‐being of the elderly by reducing the risk of complications from excessive physical activity or specific medical conditions.

**Figure 18:**
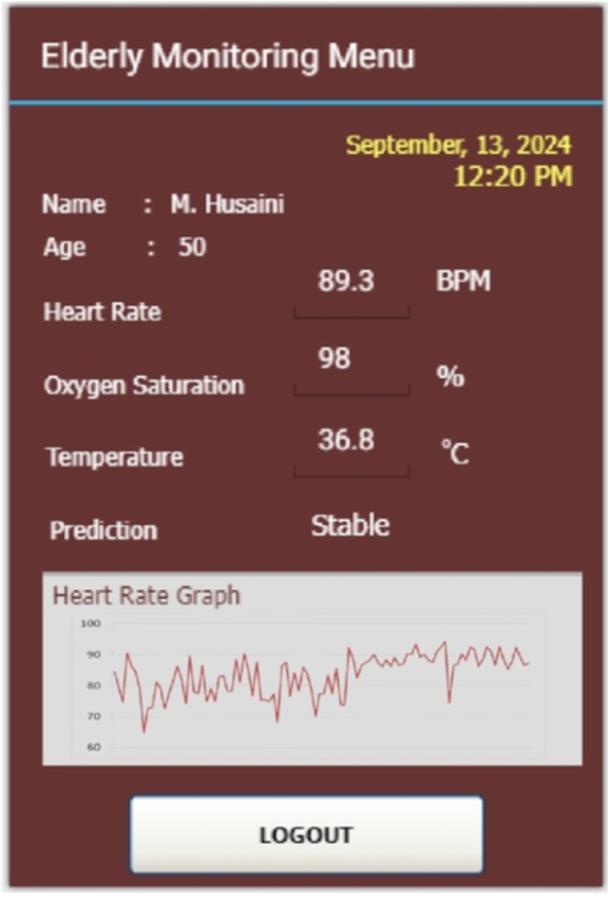
Real‐world experiment for sitting condition.

Figure 18 represents the condition of the patient in sitting condition. Data collection was carried out after participants sat for 30 min. During the initial 30 min, participants were involved in light conversation to avoid stressful conditions which had an impact on increasing parameters, especially heart‐rate parameters. So the results from reading the data are objective and optimal for sitting conditions. From the measurement results, the parameters of heart‐rate, oxygen saturation, and temperature were 89.3 BPM, 98%, and 36.8°C, respectively. If analyzed further, in the sitting condition, the heart‐rate parameter has higher results compared to sleeping condition, but has lower results compared to walking condition.

**Figure 19:**
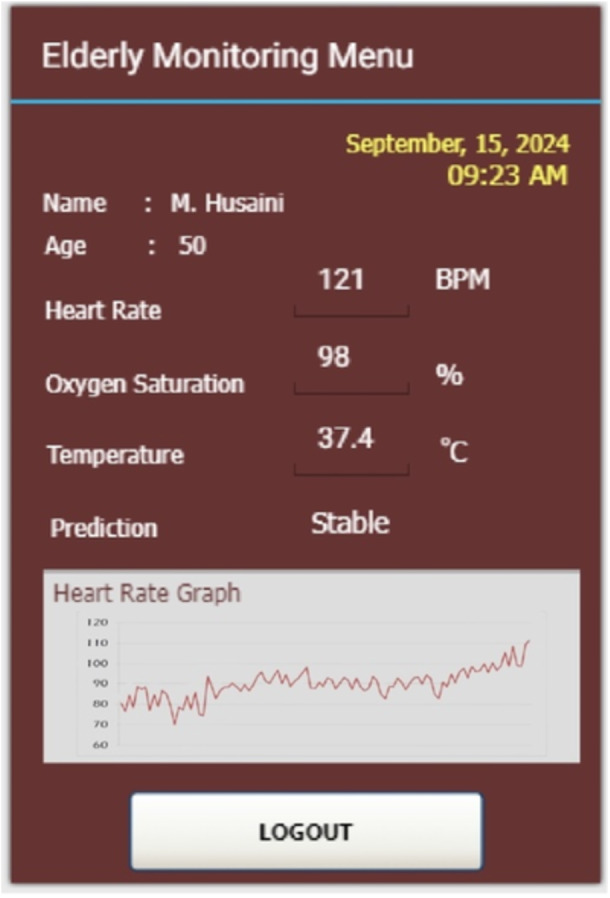
Real‐world experiment for cardio exercising condition.

Meanwhile, for cardio exercising condition is represented by Figure 19 When compared with sleeping, walking, and sitting conditions, the heart‐rate and temperature parameters in cardio exercise parameters experienced values of 121 BPM and 37.4°C, respectively. The parameter of oxygen saturation in low conditions compared to other conditions with a percentage result of 98%. The results of this experiment indicate that the system can capture the real‐world data effectively where users can easily find out the current conditions of the subjects being observed.

#### 4.3.3 User Satisfaction and Acceptance Among Elderly User and Caregivers

To find out the quality and performance of the elderly monitoring system in the user aspect, an analysis of user satisfaction to the elderly users and caregivers was carried out which focused on the criteria of user usability, comfort, security, and effectiveness. There were 8 questionnaires given to 10 participants which were defined by 5 elderly users with an age range of 50–60 years and five caregivers. The assessment scale is in the form of a Likert scale with a rating range of 1 to 5 where the lowest value is the value of 1 and the highest value is the value of 5. The mechanism for filling out the questionnaire is carried out by distributing the questionnaire after the user uses the application. This aims to fill in the questionnaire data objectively. Table 19 represents the 8 questionnaires list and categories used in user satisfaction evaluation.

**Table 19:** User satisfaction evaluation questionnaire list.

ID	Questionnaire	Category
Q1	How convenient is it to use the elderly monitoring system on a regular basis?	Usability
Q2	How easy (read: user‐friendly) is the elderly monitoring system to utilize and process the data?	
Q3	How comfortable do you feel utilizing the elderly monitoring system?	Comfort
Q4	Does the information in elderly monitoring menu is informative?	
Q5	Do you feel secure and safe when using the elderly monitoring system?	Security
Q6	Do you have any concerns about your privacy when using the monitoring system?	
Q7	How effective does the elderly monitory system provide useful information on elderly conditions?	Effectiveness
Q8	How satisfied is the elderly monitoring system?	

**Figure 20:**
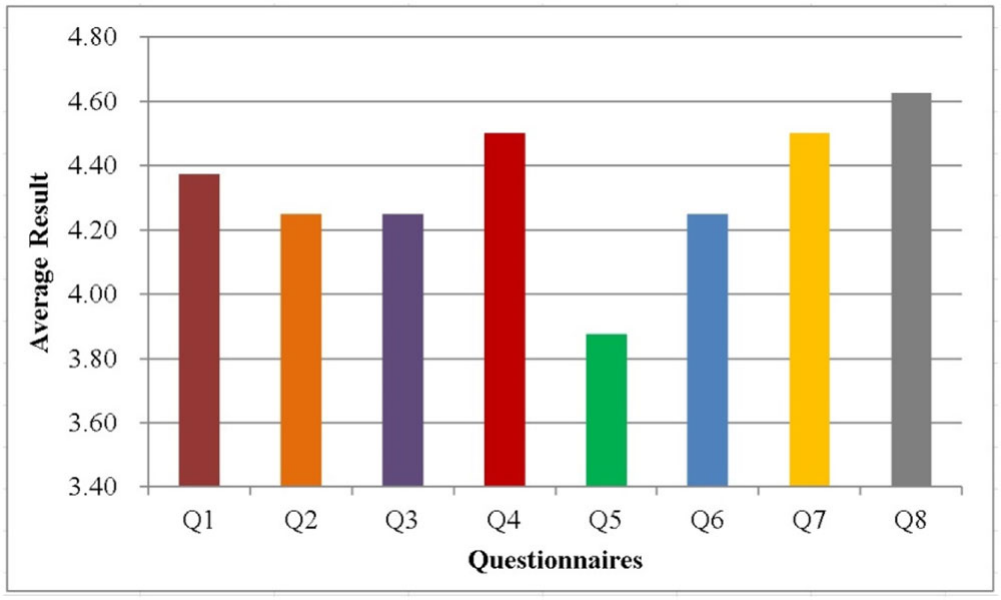
The average result for each questionnaire.

The results of the participants' questionnaires are then recapitulated and averaged to find out the value of each questionnaire. Figure 20 represents the results of the average questionnaire result where there are eight questionnaires Q1 to Q8. In these 8 questionnaires, the highest score is in Q8 with a value of 4.63 and the second highest score is in Q4 which has a value of 4.50. Meanwhile, the lowest score is found in Q5 which has a value of 3.88.

**Figure 21:**
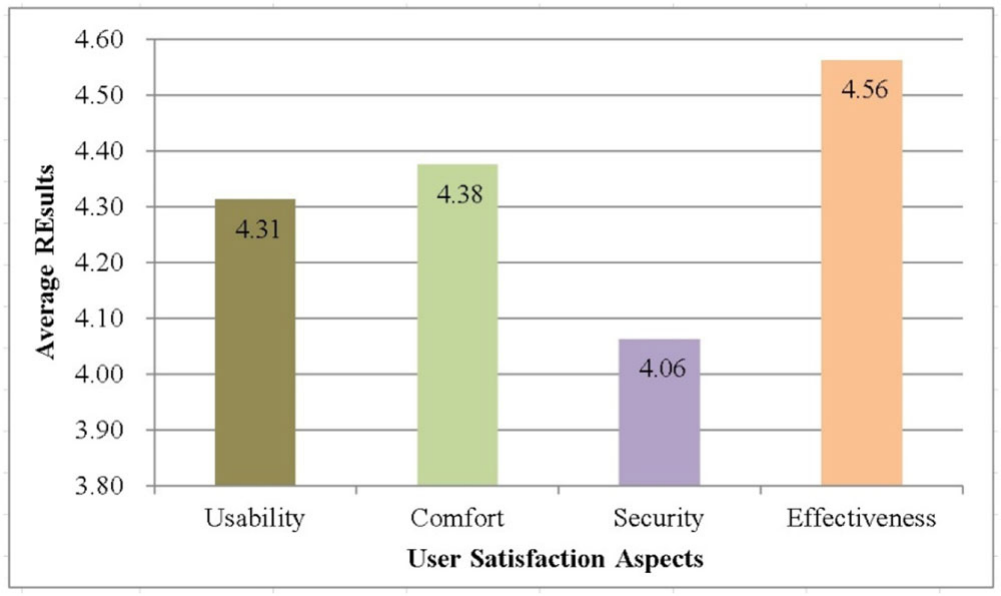
The average result for user satisfaction aspects.

Figure 21 represents the average result for each user satisfaction aspects including usability, comfort, security, and effectiveness. Overall, the score resulting from the four user satisfaction aspects is greater than value of 4 with the highest score found in the effectiveness aspect which has a value of 4.56. The lowest score is in the security aspect with a value of 4.06. Meanwhile, usability and comfort have scores of 4.31 and 4.38, respectively. Table 20 represents the user satisfaction percentage results in the four aspects where overall the average percentage result of 86.55% is obtained. This indicates that the elderly monitoring system has good performance in user satisfaction aspects. This indicates that the elderly monitoring system has good performance in user satisfaction aspects [32, 33]. added that effective and responsive monitoring is essential to ensure the safety and comfort of the elderly, and the use of IoT‐based technologies can improve user satisfaction by providing accurate and real‐time data, enabling faster and more appropriate medical interventions.

**Table 20:** User satisfaction percentage result.

Parameter	Score	Percentage
Usability	4.31	86.20%
Comfort	4.38	87.60%
Security	4.06	81.20%
Effectiveness	4.56	91.20%
**Average**	**4.33**	**86.55%**

### 4.4 Contribution, Challenge, and Limitation

Based on the experimental and discussion above the highlighted variation and contribution of this study is represented in Table 21 where there are four aspects that are the focus of contribution including accuracy performance, innovation, user satisfaction, and user convenience.

**Table 21:** The highlighted contribution of the study.

Aspect	Contribution
Accuracy performance	System development is carried out systematically to detect users through three parameters including heart‐rate, oxygen saturation, and body temperature. Validation is carried out through comparative experiments with measurement tools which produce an error percentage as an indicator of the gap between the developed system and the measurement tools. The results of the validation are in the form of a mean absolute percentage error with a percentage value of 0.90%, which in the context of accuracy indicates an accuracy percentage of 99.10%. This represents that the developed tool has a high‐accuracy performance
Innovation	The innovation implemented in the device are provide real‐time data and deep learning model to perform prediction tasks based on parameters read by the sensor. The XGBoost model is embedded into the proposed system to perform classification tasks whether the user is in a “stable” or “not stable” condition based on real‐time parameters.
User satisfaction	To validate the user perspective, an analysis of user satisfaction and acceptance among elderly users and caregivers was carried out. Four aspects are involved in user satisfaction parameters which consist of usability, comfort, security, and effectiveness. Based on the validation represents that the system device has good performance related to user satisfaction, which gains the average percentage user satisfaction of 86.55%
User convenience	The developed system has a user‐friendly interface that can be understood by general users. The graphic facilities and real‐time condition indicators of the three parameters make it easy to find out the current condition of the user.

The challenge in this research is that analyzing system behavior in various conditions is very important to understand how health monitoring systems with sensors function in real situations. For example, sensors must be able to provide accurate data both when the user is resting and during physical activity. Improvements in health monitoring and emergency response can be achieved by integrating more advanced algorithms and additional sensors that can detect various health parameters such as heart‐rate, blood oxygen levels and physical activity. Thus, this system can provide early warning and faster response in emergency situations, which is especially important for the elderly population.

However, there are several technical and operational challenges that need to be overcome. One of the main limitations is the dependence on battery power, which can limit the duration of continuous monitoring. In addition, the accuracy of the data collected by smartwatches may not be comparable to that of professional medical devices, which may affect the reliability of diagnosis. Other challenges include privacy and data security concerns, as well as the need to ensure that these devices are easy to use for parents who may not be as familiar with technology. Nevertheless, compared to traditional health monitoring systems, smartwatches offer advantages in terms of portability and convenience, which makes them an attractive option for daily health monitoring.

Recent research [34, 35, 36] indicates that in the future, the accuracy of health monitoring data can be enhanced by optimizing machine learning algorithms. Machine learning allows the system to continuously learn and adapt to user behavior, improving predictions related to changes in an individual's physical condition. For instance, machine learning models can be trained to recognize specific patterns from sensor data associated with particular health conditions, such as heart attacks or blood pressure fluctuations. By integrating these techniques, health monitoring systems can provide more timely and responsive diagnoses, ultimately improving the quality of care that can be offered. In the long run, these advancements are expected to address some of the limitations currently faced in the implementation of health monitoring technologies, especially for elderly populations.

## 5 Conclusion and Future Work

In the study, an IoT‐based elderly monitoring system device has been developed which is designed in systematic stages and involves users in the development process. In the aspect of device validation in reading the condition of elderly people, the MAPE percentage of 0.40% was obtained with detailed MAPE for heart‐rate, oxygen saturation, and body temperature parameters being 0.23%, 0.63%, and 0.33%, respectively. Development of an Android‐based user interface and integration with the Firebase cloud system is an important part of system performance. User satisfaction aspect evaluation consisting of usability, comfort, security, and effectiveness carried out on elderly users and caregiver participants resulted in a user satisfaction percentage of 86.55%. The elderly monitoring system can be implemented in practical applications by both elderly users and caregivers to see the real‐time conditions of elderly people whom they are observing. For further development, the system can be integrated with artificial technology such as machine learning, robotics, and deep learning to produce an intelligent system that can predict the condition of elderly people through data patterns resulting from the process of reading data by monitoring system devices.

## Author Contributions

**Adhan Efendi:** data curation, formal analysis, investigation, methodology, writing–original draft. **Muhammad Imam Ammarullah:** project administration, resources, visualization, validation, writing–review and editing. **Indra Griha Tofik Isa:** conceptualization, methodology, supervision, writing–review and editing. **Meli Puspita Sari:** project administration, visualization, writing–review and editing. **Jasmine Nurul Izza:** project administration, software, writing–review and editing. **Yohanes Sinung Nugroho:** project administration, resources. **Hamid Nasrullah:** project administration, resources, writing–review and editing. **Denny Alfian:** project administration, resources, writing–review and editing.

## Consent

The authors have nothing to report.

## Conflicts of Interest

The authors declare no conflicts of interest.

## Transparency Statement

The authors affirm that this article is an honest, accurate, and transparent account of the study being reported; that no important aspects of the study have been omitted; and that any discrepancies from the study as planned (and, if relevant, registered) have been explained.

## Data Availability

The necessary data used in the article are already present in the article.
